# Identification of Virulence Factors Involved in a Murine Model of Severe Achromobacter xylosoxidans Infection

**DOI:** 10.1128/iai.00037-23

**Published:** 2023-05-31

**Authors:** Brandon M. Wills, Preeti Garai, Molly O. Riegert, Felix T. Sanchez, Adam M. Pickrum, Dara W. Frank, Kenneth L. Brockman

**Affiliations:** a Department of Microbiology and Immunology, Medical College of Wisconsin, Milwaukee, Wisconsin, USA; b Department of Microbiology, New York University Grossman School of Medicine, New York, New York, USA; University of California San Diego School of Medicine

**Keywords:** *Achromobacter*, murine lung infection model, transposon mutants, CFTR, host response

## Abstract

Achromobacter xylosoxidans (Ax) is an opportunistic pathogen and causative agent of numerous infections particularly in immunocompromised individuals with increasing prevalence in cystic fibrosis (CF). To date, investigations have focused on the clinical epidemiology and genomic comparisons of Ax isolates, yet little is known about disease pathology or the role that specific virulence factors play in tissue invasion or damage. Here, we model an acute Ax lung infection in immunocompetent C57BL/6 mice and immunocompromised CF mice, revealing a link between *in vitro* cytotoxicity and disease in an intact host. Mice were intratracheally challenged with sublethal doses of a cytotoxic (GN050) or invasive (GN008) strain of Ax. Bacterial burden, immune cell populations, and inflammatory markers in bronchoalveolar lavage fluid and lung homogenates were measured at different time points to assess disease severity. CF mice had a similar but delayed immune response toward both Ax strains compared to C57BL/6J mice. GN050 caused more severe disease and higher mortality which correlated with greater bacterial burden and increased proinflammatory responses in both mouse models. In agreement with the cytotoxicity of GN050 toward macrophages *in vitro*, mice challenged with GN050 had fewer macrophages. Mutants with transposon insertions in predicted virulence factors of GN050 showed that disease severity depended on the type III secretion system, Vi capsule, antisigma-E factor, and partially on the ArtA adhesin. The development of an acute infection model provides an essential tool to better understand the infectivity of diverse Ax isolates and enable improved identification of virulence factors important to bacterial persistence and disease.

## INTRODUCTION

Members of the genus *Achromobacter* are Gram-negative, opportunistic pathogens that are widely distributed in soil and water environments. Achromobacter xylosoxidans (Ax) was initially isolated from patients with chronic otitis media (1). Since its initial isolation and preliminary biochemical characterization, Ax has been associated with serious acute and chronic infections. Individuals at risk include immunocompromised patients and those with chronic debilitating conditions, such as cancer, diabetes, renal disease, or lung disease. Ax infects a variety of sites, including soft tissues, bone, wound, skin, eye, middle ear, urinary tract, gastrointestinal tract, lower respiratory tract, bloodstream, and central nervous system, suggesting that it has the metabolic capacity to persist in different environments while potentially resisting host immune responses (2, 3). In addition to causing acute infections, Ax is considered an emerging pathogen in patients affected by cystic fibrosis (CF) (4). The increase in prevalence of Ax is likely due to greater recognition, better diagnostics, and the increase in life span of these individuals (5). Although colonization with Ax is considered a marker of severity in CF patients, the mechanisms and gene products mediating persistence and lung pathology have not been established (5).

Comparative genomics has been the primary tool used to identify potential Ax virulence genes associated with host pathology. Comparison of environmental, commensal, and CF isolates identified gene clusters associated with colonization of the CF lung that included a type III secretion system (T3SS), a polysaccharide island of capsular and cellulose synthesis genes, putative toxins, adherence-related proteins, and a region including genes involved in alcaligin biogenesis (6). Genomic and phenotypic analysis of the CF isolate NH44784-1996 identified loci encoding four potential types of secretion systems (T2, T3, T6 and T7SS), genes that mediate cell-to-surface adhesion (*pgaABCD*), denitrification (*nar*, *nir*, *nor*, and *nos* operons), and antibiotic resistance (modification enzymes and several efflux pumps) (7). Functional testing of Ax demonstrated the ability of the bacterium to be motile, produce biofilms, grow anaerobically, and possess an antibiotic resistance profile consistent with the predicted genetic composition of NH44784-1996. More recent genomic studies (8–16) and *in vitro* functional studies (17) have enabled the identification of virulence loci that are postulated to contribute to the colonization and pathology of human tissues. Notably, the role of these loci during infection has not been confirmed.

Several contributing factors have limited the progress of understanding the role of Ax in human disease, including the lack of standardized, genetically tractable strains. The DNA composition of Ax is variable and may contain mobile elements such as plasmids, phages, insertion sequences, integrative and conjugative elements, as well as hypermutator loci (9, 13, 15). Variation of different loci have an unknown impact on gene expression making transcriptional and translational analyses a necessary part of experimental design. Thus, studies involving Ax pathology generally lag since there are few reagents and genetic tools optimized for this bacterium. As a result, there are no well-characterized laboratory strains with both defined genotypes and phenotypes. To exacerbate the issue, infection models are not well-developed from the aspect of bacterial replication and host pathology. To address this gap, we previously developed a reproducible tissue culture model utilizing relatively low multiplicities of infection. We identified macrophages as an important target cell for Ax that requires bacterial uptake to induce cytotoxicity. When tested in this model, Ax clinical isolates produced a spectrum of cytopathic responses (18). Additionally, some Ax isolates appear to subvert the endocytic pathway and exist within macrophages for an extended period of time with limited ability to replicate. However, it is unknown if these same responses occur *in vivo*.

Here, we develop an *in vivo* murine model of infection for Ax using two sequenced isolates that have opposing cytotoxicity profiles toward macrophages *in vitro*. These studies aim to quantify bacterial maintenance or replication of strains with different *in vitro* pathologies. Additionally, the host response to each type of strain is assessed. We test the validity of this model by measuring attenuation of transposon insertion mutations in a cytotoxic strain. These analyses represent an important step in identifying and characterizing Ax virulence factors and fitness determinants used *in vivo*.

## RESULTS

### Severity of experimental disease correlates with AX *in vitro* phenotypes.

**FIG 1:**
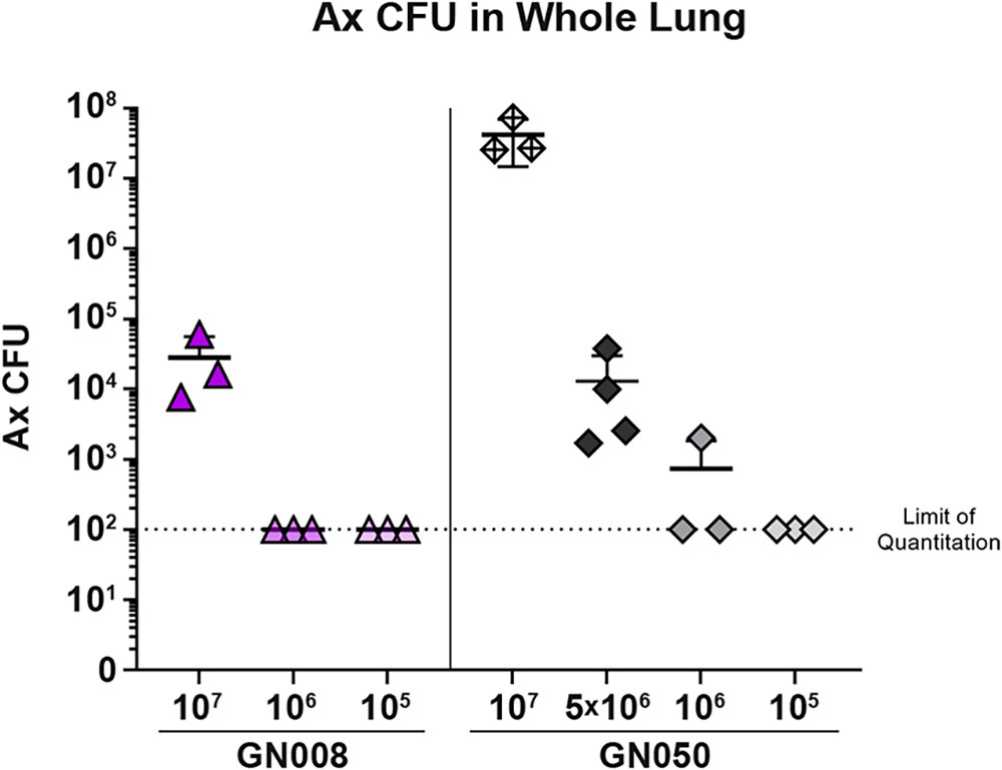
Determination of optimal challenge doses in CF mice. Total CFU of Ax in lungs of Cftr^−/−^ mice infected with different doses of GN008 or GN050 for 96 h. *n* = 3 or 4, as indicated by the symbols.

To begin to understand the infectivity and disease pathology of the different Ax *in vitro* phenotypes, we took advantage of strains previously assessed by our group. Ax GN008 is characterized as nonacutely cytotoxic and potentially invasive to macrophages *in vitro* (18, 19). Ax GN050 is an acutely cytotoxic isolate capable of inducing rapid macrophage lysis *in vitro* (18). CF mice that lack the cystic fibrosis transmembrane conductance regulator (CFTR) protein (CFTR S489X-; FABP-hCFTR) were infected intratracheally with either 10^7^, 10^6^, or 10^5^ Ax CFU and monitored daily for up to 4 d (96 h) for signs of distress and morbidity. On day 4 postinoculation, mice were euthanized and then bronchoalveolar lavage (BAL) fluids and whole lung homogenates were collected to enumerate bacterial burden within the lungs. Mice challenged with the greatest dose of the cytotoxic strain, 10^7^ GN050, became moribund by 48 h postinfection and were euthanized. The number of Ax within the lungs of these mice was four times the initial inoculum dose (4.2×10^7^, Fig. 1), which likely contributed to morbidity. Mice challenged with 10^6^ or 10^5^ GN050 had completely or nearly cleared Ax from the lungs by 96 h postinoculation. Based on the wide variance between the 10^7^ and 10^6^ doses for GN050, we tested an additional dose of 5×10^6^ CFU GN050. All four of the mice infected with 5×10^6^ CFU GN050 survived and were culture positive after 96 h. In contrast to mice infected with GN050, all the mice challenged with GN008, the strain invasive to macrophages, survived to 96 h postinfection. The 3 mice challenged with 10^7^ CFU GN008 were positive for Ax in the lungs, with a greater than 2-log reduction in total CFU (2.8×10^4^, Fig. 1) at 96 h compared to the challenge inoculum. All the mice challenged with 10^6^ or 10^5^ GN008 had cleared the bacteria from the lungs by 96 h (Fig. 1). Based on these dose response studies, challenge doses of 5×10^6^ for GN050 and 10^7^ for GN008 were selected for further study as they resulted in a nonlethal infection in which the lungs remained culture-positive for at least 96 h.

**FIG 2:**
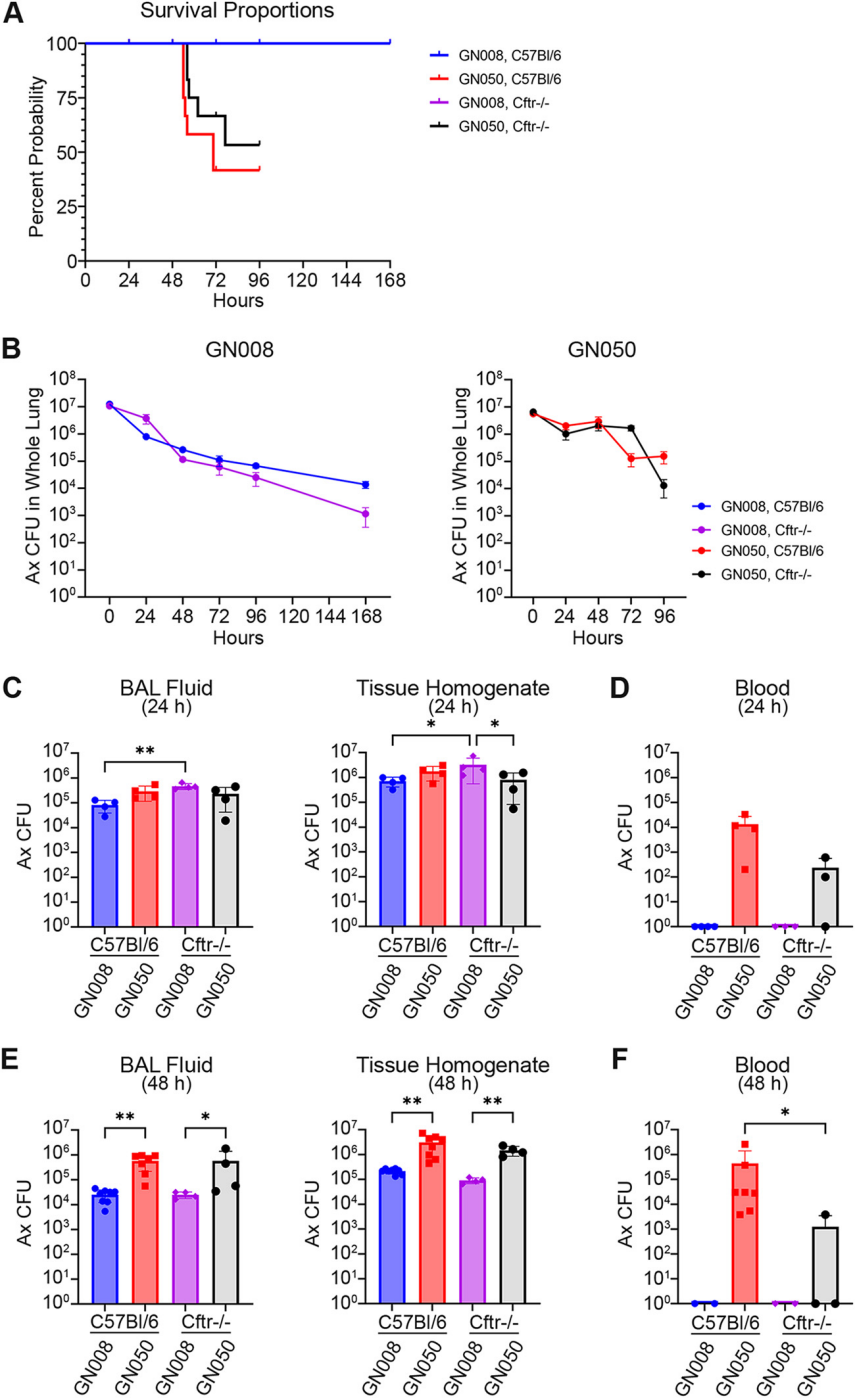
Bacterial burden and persistence of Ax in the lungs of C57BL/6 and CF mice. C57BL/6 and Cftr^−/−^ mice were infected with 10^7^ GN008 or 5 × 10^6^ GN050. (A) Survival of infected mice. (B) Total CFU in the lungs of infected mice over the course of infection. (C) Ax CFU in the BAL fluids or lung tissues 24 h after infection. *n* = 4. (D) Ax CFU in the blood 24 h after infection. *n* = 3 to 4. (E) Ax CFU in the BAL fluids or lung tissues 48 h after infection. *n* = 8 for C57BL/6; *n* = 4 for Cftr^−/−^. (F) Ax in the blood 48 h after infection. *n* = 3 to 7. *, *P* < 0.05; **, *P* < 0.01. One-way ANOVA.

To define the bacterial burden and host immune response within the lungs over time, mice were challenged with 5×10^6^ CFU GN050 or 10^7^ CFU GN008 via intratracheal instillation. Cohorts of 20 CF mice or 20 wild-type C57BL/6 mice were infected and monitored daily for signs of disease. Predetermined groups of 4 mice (2 male & 2 female) were euthanized at 24, 48, 72, 96, or 168 h postinoculation. Mice challenged with GN008 all survived until their cohort’s designated day of euthanization. None of the mice infected with GN050 died or were euthanized in the first 48 h. However, one C57BL/6 and one CF mouse from the GN050 72 h groups died or was euthanized prior to 72 h. Due to severe morbidity, mice challenged with GN050 and designated to be euthanized at 168 h were euthanized early and combined with the 96-h group for a total of 8 potential mice for the final time point. Half of the CF mice (4 of 8) and one quarter of the C57BL/6 mice (2 of 8) survived to 96 h. The median survival for mice infected with GN050 was 77 h with a hazard ratio of 0.097 compared to mice infected with GN008 (Fig. 2A; *P* < 0.0001, Mantel-Haenszel). Samples collected from mice that died prior to their designated time point were excluded from additional analyses.

At the time of euthanasia, bronchoalveolar lavage was performed and tissues from whole lungs were homogenized to determine bacterial burden. Mice challenged with GN008 had a moderate decrease in Ax within the lungs at 24 h, followed by a steady decrease in Ax burden over the course of 7 d. All mice challenged with GN008 still contained Ax within their lungs 168 h postinfection, the last time point assessed. Following an initial decrease in CFU at 24 h, the burden of GN050 within the lungs peaked at 48 h and remained relatively constant in the lungs of CF mice for the first 72 h. For CF mice that survived to 96 h (4 of 8) the number of GN050 within the lungs decreased approximately 2 logs from the 72 h to 96 h time points (Fig. 2B). The largest difference in bacterial burden between Ax strains occurred 48 h after infection wherein GN050 had significantly greater burden in both the BAL fluids and lung tissues compared to GN008, regardless of mouse genotype (Fig. 2E). Overall, the patterns of Ax CFU in mouse tissues suggest that the cytotoxic strain, GN050, was able to maintain higher bacterial numbers early during infection, indicating replication. In contrast, GN008, the strain invasive to macrophages, was slowly reduced over time but persisted to 7 d. The bacterial burden of GN008 was significantly greater in Cftr^−/−^ mice compared to C57BL/6 mice at 24 h (Fig. 2C). This trend appeared to change by 48 h, although none of the later time points were statistically different. These results suggest an initial delay in bacterial clearance by the CF mice (Fig. 2E).

To assess potential dissemination from the lungs, blood was collected at 24 h and 48 h after intratracheal challenge with GN008 or GN050. None of the mice infected with GN008 had detectable Ax in the blood. In contrast, GN050 was detected in the blood of all C57BL/6 mice at 24 h and 48 h (Fig. 2D and F). C57BL/6 mice had a greater concentration of Ax in the blood compared to Cftr^−/−^ mice, of which only half of the mice had viable Ax in the blood (Fig. 2D and F).

### Ax with different *in vitro* phenotypes elicited a unique immune profile within the lungs.

**FIG 3:**
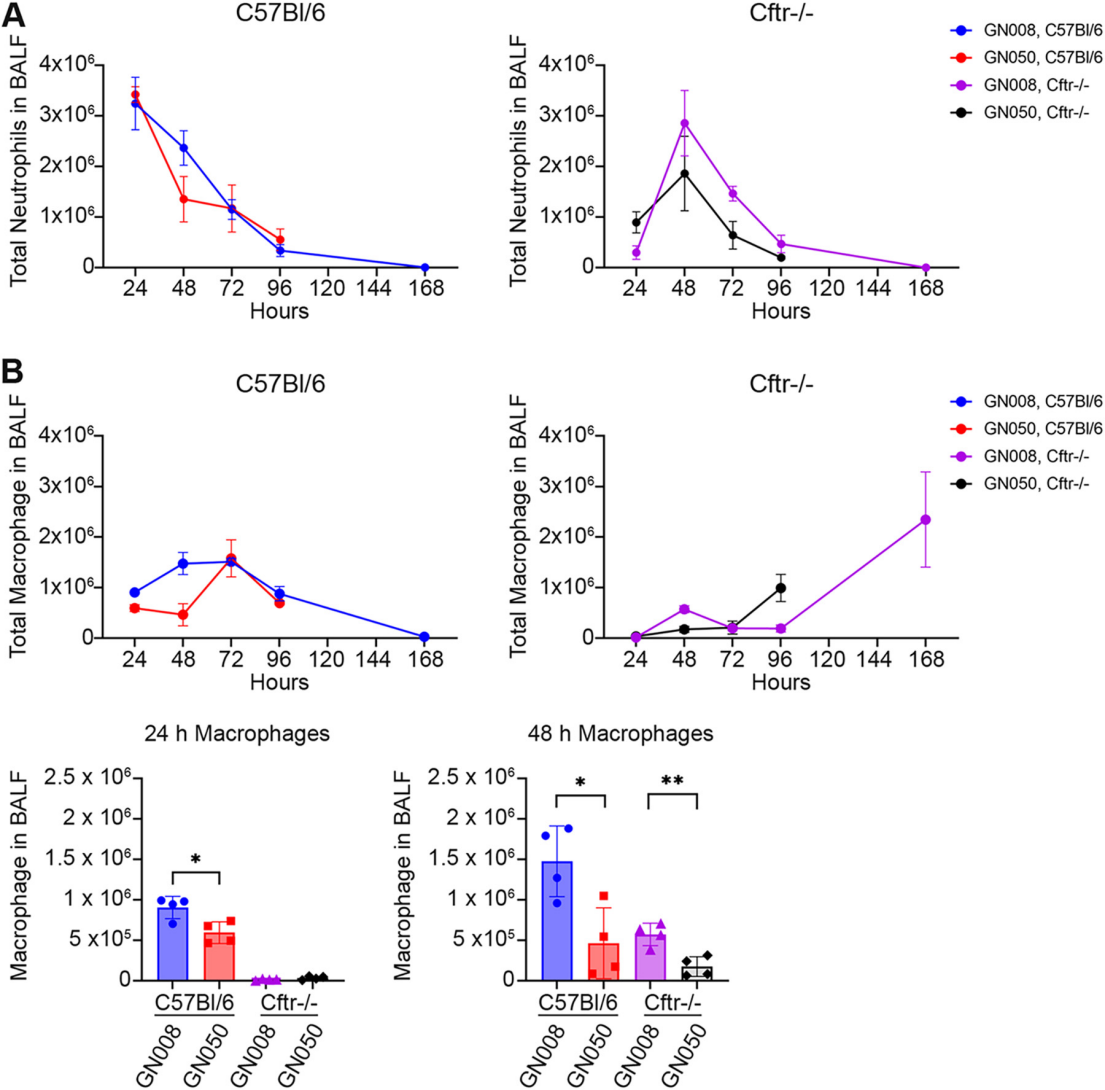
Immune cell infiltration into the lung over the course of infection. Immune cells within the BAL fluids were analyzed by flow cytometry based on surface markers. (A) Neutrophils (CD45^+^ CD11b^+^ Ly-6G^+^) in the BAL fluid. (B) Macrophages (CD45^+^ CD11b^+^ F4/80^+^) in the BAL fluids. *n* = 4. *, *P* < 0.05; ***, *P* < 0.005. One-way ANOVA.

Innate immune response within the lungs is critical to bacterial clearance and disease pathology during infection. To begin to assess immune response within the lungs during acute Ax infection with cytotoxic and invasive phenotypic strains, immune cells within the BAL fluids were analyzed by flow cytometry to quantify neutrophils (CD45^+^ CD11b^+^ Ly6G^+^) and macrophages (CD45^+^ CD11b^+^ F4/80^+^) (see Fig. S1 in the supplemental material). Infected C57BL/6 mice had a robust neutrophil response by 24 h that slowly declined over time, regardless of the phenotype of the Ax strain used for infection. In contrast, CF mice infected with either Ax strain exhibited a delayed neutrophil response with a peak infiltration at 48 h. Statistically significant differences in neutrophil infiltration were not observed between Ax strains; however, GN008 infection tended to induce greater infiltration at 48 h (Fig. 3A). For both Ax strains, macrophage infiltration peaked at 72 h in infected C57BL/6 mice. Interestingly, C57BL/6 mice infected with GN050 had significantly fewer macrophages at 24 and 48 h compared to those infected with GN008. CF mice had less macrophages compared to C57BL/6 mice, but CF mice infected with GN008 again had significantly more macrophages within the lungs at 48 h compared to those infected with GN050 (Fig. 3B). Decreased infiltration or greater cell death could contribute to the reduced number of macrophages in animals infected with GN050. We observed a second increase in macrophages at 96 h and 168 h for CF mice infected with GN050 and GN008, respectively. This secondary wave of infiltration could be due to bacterial persistence, or these cells may be a functionally different type of macrophage. Based on these data, Ax induces a similar innate cellular immune response in both mouse genotypes with a delayed response in CF mice compared to C57BL/6. Furthermore, the lungs of mice infected with the cytotoxic GN050 had significantly fewer live macrophages compared to those infected with GN008, the strain invasive to macrophages.

**FIG 4:**
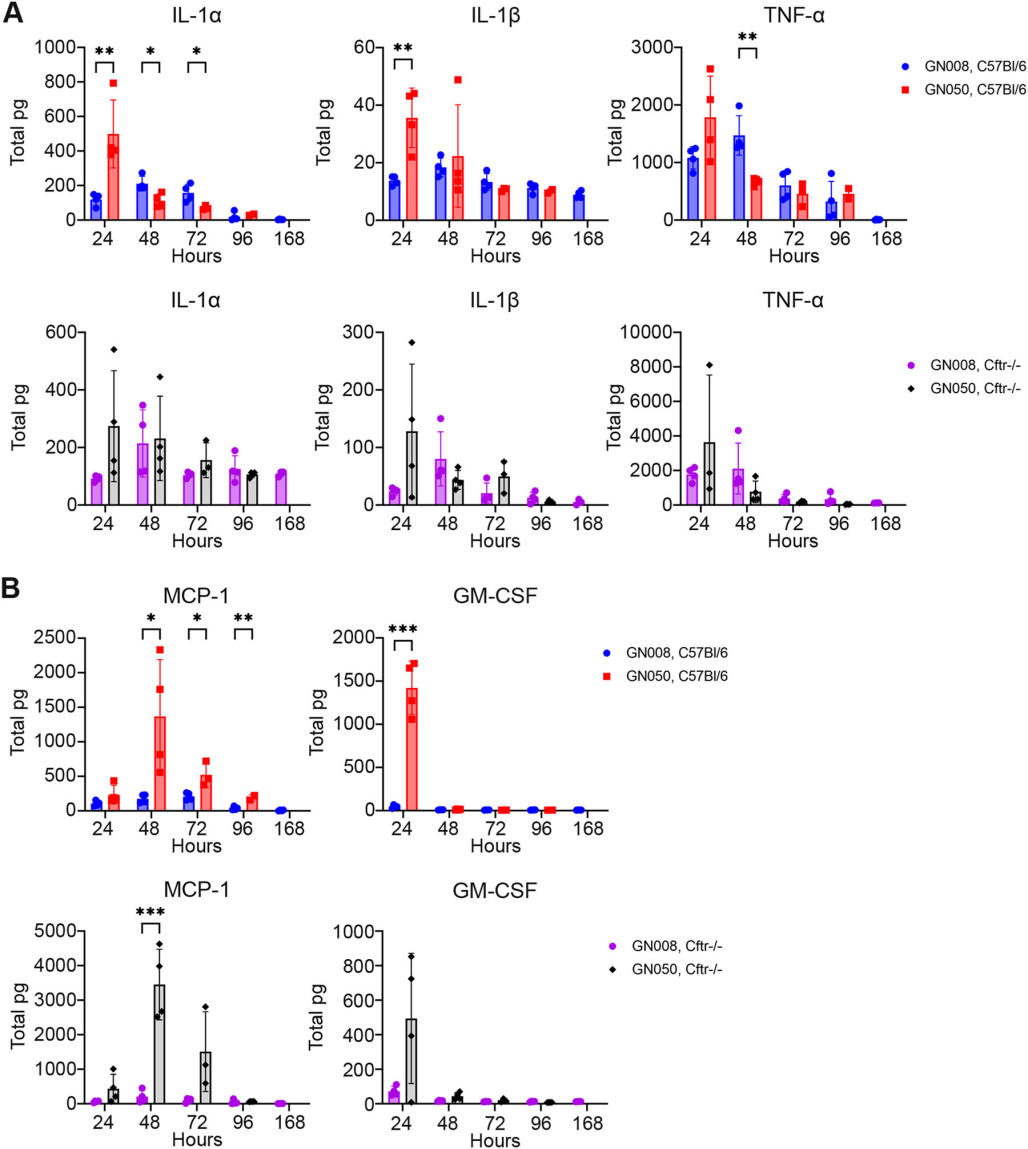
Cytokines within the BAL fluids of Ax infected mice. Absolute cytokine levels within the BAL fluids collected from C57BL/6 and Cftr^−/−^ mice infected with Ax GN008 or GN050. (A) Levels of proinflammatory cytokines IL-1α, IL-1β, and TNF in the BAL fluids. (B) Levels of MCP-1 and GM-CSF in the BAL fluids. pg, picograms. *n* = 4. *, *P* < 0.05; **, *P* < 0.01; ***, *P* < 0.005; Student's *t* test.

In addition to immune cell infiltration, inflammatory cytokine protein levels within the BAL fluids were measured. C57BL/6 mice infected with GN050 had the greatest proinflammatory cytokine concentrations at the earliest time point tested (24 h), and significantly more interleukin-1 alpha (IL-1α) and beta (IL-1β) than those infected with GN008 (Fig. 4A). In contrast, mice infected with GN008 had peak cytokine levels at 48 h and had significantly greater IL-1α and tumor necrosis factor (TNF) concentrations compared to GN050-infected mice at this time point. Similar trends in proinflammatory cytokine levels were observed with the CF mice but were not statistically significant (Fig. 4A). In general, concentrations of proinflammatory cytokines correlated with bacterial abundance, as would be expected for a Gram-negative pathogen within the lungs. Interestingly, there was an abundant production of soluble MCP-1 and GM-CSF in both C57BL/6 and CF mice infected with GN050 but not in those infected with GN008. MCP-1 levels in the lungs of mice infected with GN050 peaked at 48 h and returned to basal levels over the next 48 h. GM-CSF levels, in contrast, peaked at 24 h following GN050 infection and then rapidly returned to basal levels by 48 h (Fig. 4B). No significant increase in MCP-1 or GM-CSF was observed in mice infected with GN008. These data suggest that GN050 induces a unique response that may promote the infiltration, proliferation, and maturation of myeloid cells, specifically monocytes and macrophages.

### Ax infection and lung pathology.

**FIG 5:**
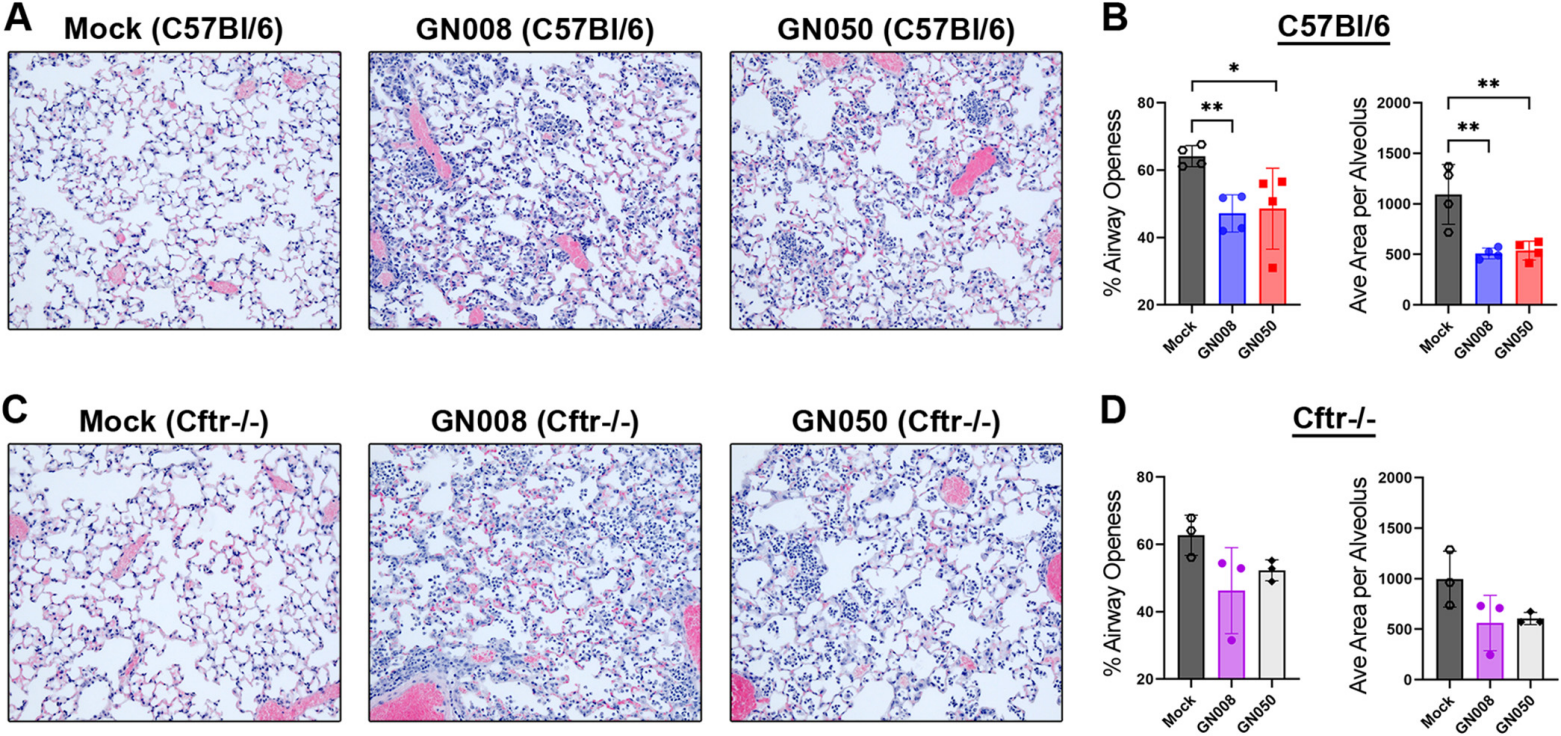
Histological analysis of infected lungs from C57BL/6 and CF mice. (A) Representative hematoxylin and eosin (H&E) stained images of saline, GN008, or GN050 infected lungs of C57BL/6 mice. (B) Percent airway openness and average area of airway space of mock-infected (saline), GN008-, or GN050-infected lungs of C57BL/6 and Cftr^−/−^ mice. (C) Representative H&E images of saline, GN008-, or GN050-infected lungs of Cftr^−/−^ mice. (D) Percent airway openness and average area per alveolus of saline, GN008-, or GN050-infected lungs of Cftr^−/−^ mice. *n* = 4 for C57BL/6, *n* = 3 for Cftr^−/−^. *, *P* < 0.05; **, *P* < 0.01; One-way ANOVA.

Immune cell infiltration and overall lung pathology was assessed on hematoxylin and eosin (H&E) stained sections of paraffin embedded lungs that had been infected with Ax for 48 h. Mock-infected mice were intratracheally instilled with sterile saline diluent. The lungs of mock-infected C57BL/6 and CF mice appeared to have alveoli that were clear and had minimal to no immune cell infiltration. Approximately 63% of the airways were open, or air-filled, based on average cell-free area across the field of view. Ax-infected lungs had increased edema, regions of alveolar wall thickening, and significant immune cell infiltration compared to mock-infected lungs (Fig. 5A and C). Lungs of infected C57BL/6 mice had a significant decrease in overall airway openness and average alveolar area, predominantly due to the accumulation of infiltrating polymorphonuclear cells (PMNs) (Fig. 5B). A similar trend was observed with the CF mice, but the results appeared to be more variable in these animals (Fig. 5D). Taken together, our results indicate a strong inflammatory response within the lungs during acute infection and a potential Ax strain-dependent macrophage response.

### Analysis of GN050 derivatives with transposon insertions.

**FIG 6:**
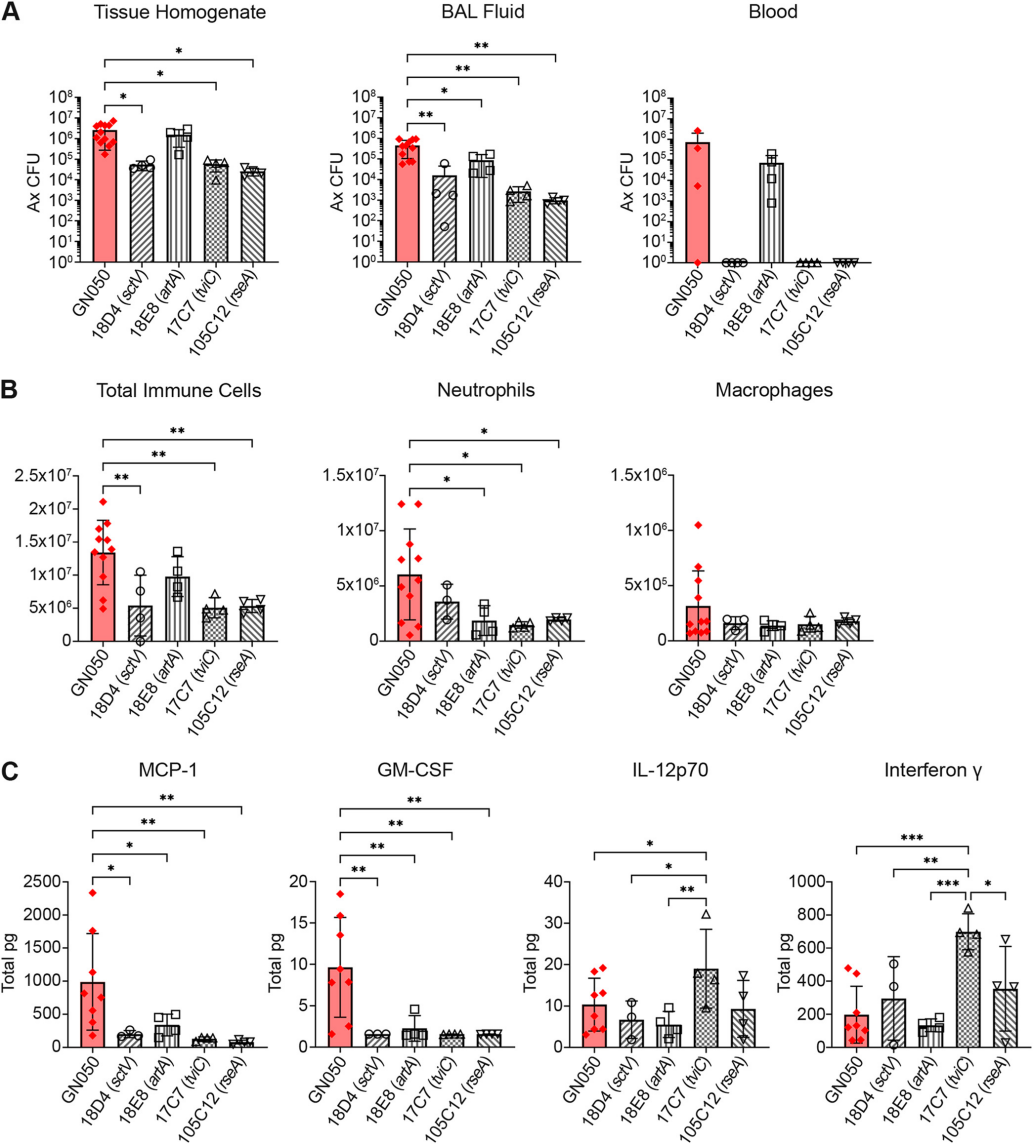
Bacterial burden and immune response in C57BL/6 mice infected with GN050 *himar* mutants. C57BL/6 mice infected with the GN050 parent strain or *himar*1 insertion mutants for 48 h. The mutants used were 18D4 (*sctV*), 18E8 (*artA*), 17C7 (*tviC*), and 105C12 (*rseA*). (A) Ax CFU in the lung tissue, BAL fluid, and blood. (B) Total immune cells, neutrophils, and macrophages in the BAL fluids. (C) Cytokine levels in the BAL fluids. pg = picograms. *n* = 8 for GN050, and *n* = 4 for *himar* mutants. *, *P* < 0.05; **, *P* < 0.01; ***, *P* < 0.005; One-way ANOVA.

GN050 appears to induce more severe infection and pathological responses *in vivo* compared to GN008. These findings correlate with the more acutely cytotoxic nature of GN050 in an *in vitro* model of infection (18). To begin to determine virulence factors that contribute to the unique immune response and severe pathology of GN050, a variety of transposon mutants with insertions in predicted key virulence genes (19) were tested *in vivo*. Mutants were selected based on significantly decreased cytotoxicity of macrophages *in vitro* (19). The first mutant (18D4) has an insertion located within the open reading frame (ORF) of *sctV*, which is required for a functional type III secretion system (T3SS). The next mutant (18E8) has an insertion in the gene encoding a large RTX (repeats-in-toxin) adhesin (ArtA), which has been characterized as important for *in vitro* macrophage attachment and cytotoxicity (19). Additionally, insertions in the ORFs potentially encoding products involved in Vi polysaccharide biosynthesis (17C7, *tviC*) and an antisigma-E factor that is postulated to regulate various stress responses (105C12, *rseA*) were tested. Each of these genes are present in both GN008 and GN050 and are expressed at the level of transcription under *in vitro* and *in vivo* growth conditions (Fig. S2). C57BL/6 mice were infected with 5 × 10^6^ CFU of the GN050 parent or one of four mutants. At 48 h postinfection, mice were euthanized and bacterial burden was assessed within the BAL fluids and lung tissues. The *sctV*, *tviC*, and *rseA* mutants were significantly less abundant in the BAL fluids and lungs in comparison to the GN050 parent strain (Fig. 6A). A mutant with a transposon insertion within the *artA* ORF did not significantly affect bacterial burden in the lung tissue, but did reduce bacterial CFU in the BAL fluids, although not to the same extent as the other mutants. Interestingly, the *sctV*, *tviC*, and *rseA* mutants were not detected in the blood, suggesting that T3SS, Vi capsule, and antisigma-E stress responses are directly or indirectly involved in Ax dissemination from the lungs. The *artA* mutant was able to disseminate into the blood, but at a lower concentration than that of the parental GN050 strain at 48 h (Fig. 6A). Further, none of the mice infected with the *sctV*, *tviC*, or *rseA* mutants showed signs of morbidity during the 48 h of infection, suggesting that dissemination may be a key factor in the severe pathology and mortality observed with strain GN050. Mice infected with the transposon mutants had fewer total immune cells and decreased neutrophils within their BAL fluids compared to those infected with the parent GN050 (Fig. 6B). Compared to the wild-type GN050, MCP-1 levels were significantly reduced in the lungs of mice infected with any of the transposon-bearing strains (Fig. 6C). GM-CSF was detectable in mice infected with parent GN050 at 48 h, albeit at very low levels, but concentrations were below the limit of detection in mice infected with any of the mutants (Fig. 6C). Interestingly, mice infected with the *tviC* insertion mutant had significantly increased levels of IL-12-p70 and interferon gamma compared to the other mutants and parent GN050 strain (Fig. 6C). These data suggest that infection with these *himar* mutants induces less immune response and reduced morbidity compared to the GN050 parent.

## DISCUSSION

Ax causes a variety of infections and chronic diseases, particularly in immunocompromised patients, yet there are few mechanistic studies that address the interplay of bacterial virulence factors and host responses. This is not due to a lack of attempts to develop models of infection. Hyodo et al. (20) demonstrated the role of cellular immunity in protection against systemic infection of BALB/c mice infected with a cerebrospinal isolate of Ax. In a model of inflammatory pneumonia, Prado et al. demonstrated a role for leukotriene B4 in reduction of lung edema and increased levels of α-defensin-1 that aided in the clearance of Ax (21). More recently, the role of inflammation and inflammatory signaling during Ax-mediated pneumonia in mice was addressed using CD14-deficient mice. CD14-deficient mice were protected from Ax-induced death, which was unrelated to bacterial burden (22). In general, bacterial strains used in most *in vitro* and limited *in vivo* studies are characterized only as far as the type of patient (CF), the frequency of isolation (occasional/chronic), or the tissue from which the bacterium was isolated (non-CF patients). Here, we report the development of an *in vivo* model of Ax infection with well-characterized and sequenced isolates previously shown to be either acutely cytotoxic or invasive to macrophages *in vitro*.

Experiments were carried out in both immunocompetent C57BL/6 wild-type mice as well as mice that lack proper expression of CFTR within the lung and immune cells. Due to the complexities of cystic fibrosis, there is no ideal experimental model that can recapitulate all aspects of human disease. Initial studies herein opted to use a murine model of lung infection to begin to understand the interactions and roles of Ax within the lung, as such our findings could inform studies with other CF experimental models, such as ferret or pig (23). The survival rates and bacterial burden within the lung was similar with C57BL/6 and Cftr^−/−^ mice; however, CF mice had delayed immune cell infiltration and overall decreased macrophage response. It has been reported previously that mice lacking CFTR have a defective myeloid response and increased basal lung infiltration (24). Following Pseudomonas infection, Cftr^−/−^ mice exhibit increased lung inflammation pathology and reduced bacterial clearance compared to wild-type mice (25). In wild-type C57BL/6 mice, acute lung infection with planktonic Pseudomonas is relatively short and the mice generally either clear the bacteria or succumb to infection within 3 days (26). In our model, infection with GN008, an Ax strain isolated from the sputum of a CF patient, persists through 7 days in both C57BL/6 and Cftr^−/−^ mice (Fig. 2). Several studies have shown that infection of CF mice with Staphylococcus aureus or Burkholderia cepacia results in increased bacterial burden and greater inflammation compared to wild-type counterparts (26). Reports on infection with Pseudomonas are more inconsistent and the disease outcome varies with the bacterial strain tested (26). As innate cellular immune response is critical during Ax infection, the phenotypes observed with the CF mice are likely driven by a combination of malfunctioning myeloid responses and epithelial cell defects. Studies by Gosselin et al. (27) and Heeckeren et al. (28) showed that the use of Pseudomonas embedded in agar beads could establish a more chronic lung infection in mice, and that CF mice were significantly more susceptible to chronic infection with mucoid strains than non-CF animals. In these later studies, CF mice tended to have greater proinflammatory cytokine concentrations compared to non-CF mice; however, we did not notice significant differences in proinflammatory cytokines between CF and non-CF mice infected with Ax (Fig. 4). The response of CF mice to Ax infection was more inconsistent than that of C57BL/6 mice and was not correlated with sex. In initial pilot studies, older CF mice tended to have more severe disease (data not shown), but no age-related differences were observed with the young, 8- to 12-week-old mice used for the studies presented here.

The two clinical Ax isolates used in this study were selected based on anatomical source (CF sputum versus ear) and *in vitro* phenotypes. Strain GN008 was isolated from the sputum of a CF patient and was relatively less cytotoxic to macrophages *in vitro* (18). In contrast, GN050 was isolated from the ear effusion of a patient with otitis media and was acutely cytotoxic to macrophages *in vitro*, causing cellular lysis and macrophage death within hours of infection (18). *In vivo*, GN050 was significantly more virulent than GN008, with a 50 percent lethal dose (LD50) of 5×10^6^ at 48 h. The increased lethality of this strain appears to be driven by altered immune cell responses leading to dissemination and sepsis. Following intratracheal infection, GN050 was detected in the blood of infected mice by 24 h, yet Ax was not detected in the blood of any of the mice infected with GN008 (Fig. 2D and F). CF mice had significantly less Ax in the blood, likely due to decreased dissemination, reduced survival in the blood, or both. The exact mechanisms of dissemination remain to be determined; however, an intact type III secretion system, Vi capsule expression, and antisigma-E stress responses all appear to be necessary. GN050 infection produced a greater initial proinflammatory cytokine response, yet the lungs of these mice had significantly fewer macrophages during the first 48 h compared to those infected with the nonlethal GN008. GN050 is acutely cytotoxic to macrophages *in vitro* and Ax-mediated macrophage death may drive the reduced numbers of live macrophages in the lungs. In support of this hypothesis, GN050, but not GN008, induced high levels of MCP-1 and GM-CSF within the BAL fluids. Classically, MCP-1 and GM-CSF are produced in response to elevated IL-1 and TNF, or bacterial factors such as endotoxin (29, 30); however, the significant increase of MCP-1 and GM-CSF is seen only with GN050, suggesting an alternative mechanism. MCP-1 is produced by a variety of cells, including macrophages and functions to recruit monocytes and macrophages to the site of infection (31). During Listeria monocytogenes infection, MCP-1 production requires cytoplasmic localization of bacteria (32), thus it is possible that the ability of GN050 to alter endocytic trafficking may drive increased MCP-1 production. GM-CSF stimulates the proliferation and activation of granulocytes and macrophages; however, overexpression can lead to severe inflammation (30). Infection with a variety of GN050 mutants that are severely defective for some stage of macrophage lysis *in vitro* (binding, entry, or production of a toxin) fail to induce significant MCP-1 and GM-CSF levels, resulting in less lung inflammation and an inability to disseminate into the blood. The ability to enter cells and cause a rapid cytotoxic response thus, in part, contributes to the observed disease severity caused by GN050. In contrast, the *artA* mutant (18E8), which is hampered but not defective for binding and entry, maintained intermediate bacterial loads within the lung and was still able to disseminate into the blood by 48 h. This delayed but not incomplete toxicity may contribute to the intermediate phenotypes observed with this mutant and further highlights the sensitivity of this experimental animal model to discern subtle differences in Ax phenotypes.

Intriguingly, the *tviC* mutant, which is predicted to be defective for Vi capsule synthesis, induced significantly greater IL-12 and interferon gamma (IFN-γ) compared to the parent GN050 and the other mutants (Fig. 6C). There was no apparent acute effect on immune cell infiltration or the expression of other cytokines compared to the other mutants tested. IL-12 is produced by macrophages in response to intracellular bacteria or bacterial components such as lipopolysaccharides (LPS) and stimulates the production of interferon gamma by T cells and NK cells. Interferon gamma then acts on macrophages to induce expression of MHC-II and antimicrobial products. The IL-12/IFN-γ axis is critical in the control of intracellular mycobacterium and disseminated salmonella infections (33, 34). It is possible that the Vi capsule of Ax protects against IL-12-mediated clearance, shields against immune recognition of other surface factors, or promotes binding to host cells. Future work is needed to fully understand the complex interactions of these Ax virulence factors within the host. The development of strategies to prevent the binding and entry of Ax may provide protection against severe acute and chronic infections in susceptible populations.

## MATERIALS AND METHODS

### Bacterial culture.

A. xylosoxidans GN008 and GN050 (18, 19) were obtained from MCW clinical laboratories and cultured on Luria-Bertani (LB) agar at 37°C for 1 day and then at room temperature for 1 day. For infections, bacterial emulsions were made from cultures on plates, assessed by optical density measurements, and diluted to the target inoculum CFU. Actual CFU of inocula were determined by serial dilution and plating on LB agar. Murine lung tissues, blood, and bronchoalveolar lavage fluids were treated similarly and assessed for CFU by serial dilution and plating.

### Murine infections.

C57BL/6 and Cftrtm1Unc Tg(FABPCFTR)1Jaw/J mice were obtained from The Jackson Laboratory and bred in-house under an approved animal use protocol. Cftr^−/−^ mice were genotyped using the suggested primers (The Jackson Laboratory). For intratracheal instillation, mice were anesthetized, and a blunt needle was used to deliver 100 μL of the appropriate Ax strain directly into the lungs. At the indicated time points, mice were euthanized, blood was collected, and bronchoalveolar lavage was performed, as described previously (35). Briefly, a blunt needle was inserted into the trachea and the bronchioles were lavaged twice with 1 mL 0.9% sterile saline. Fluids from sequential lavages were pooled and aliquots of the fluids were used to determine Ax CFU and total immune cell concentrations. Following bronchoalveolar lavage, whole lungs were carefully dissected from the mouse and tissues were homogenized via MACS tissue dissociation (Miltenyi Biotec). An aliquot of the homogenized lung tissues was used to determine Ax burden.

### Analysis of immune cell composition in the BAL fluids.

Bronchoalveolar lavage fluids were gently centrifuged and cell-free supernatants were snap-frozen and stored. Cells were suspended in Dulbecco’s Phosphate Buffered Saline (DPBS) with 0.1 mM EDTA, enumerated, and divided for cell specific analysis. Washed cells were stained with Zombie Green to identify dead cells and then labeled with a cocktail of the following antibodies: BV421 antimouse/human CD11b clone M1/70 (Biolegend), BV570 antimouse CD11c clone N418 (Biolegend), APC antimouse Ly-6G clone 1A8 (Biolegend), PE antimouse F4/80 clone BM8 (Biolegend), and PE-Cy7 antimouse CD45 clone QA17A26 (Biolegend). Labeled cells were analyzed on an LSR II cytofluorometer (Becton, Dickinson, BD) or AURORA spectral cytometer (Cytek). Cells were gated according to size (SSC-A) and forward scatter (FSC-A/W) to obtain cell singlets and Zombie Green (Biolegend) was used to remove dead cells. Data were analyzed with FlowJo v10.8 software (BD) and absolute cell numbers were calculated with the total cell count multiplied successively by the percentages for the appropriate gates obtained through flow cytometry.

### Murine lung histology.

Mice were challenged via intratracheal instillation as described above. At the specified time points, mice were euthanized and the lungs were processed for histological assessment, as described previously (36). Briefly, lungs were fixed with 10% phosphate-buffered formalin via a needle inserted into the trachea. Lungs were then carefully removed from the animal and submerged in 10% phosphate-buffered formalin overnight. Fixed whole lungs were processed, paraffin embedded, and 4 μm transverse sections were taken from the middle of the lungs. Hematoxylin and eosin (H&E) staining was performed, and the stained sections were imaged on an Axioscope 5 microscope with Zen 3.4 (Zeiss). Two representative images for each mouse were collected using a 20× objective. Percent airway openness (percent field of view containing no cells or tissue) and average area per alveolus were calculated using the analyze particles feature of ImageJ 1.53 (NIH). Data points represent the average value per mouse, from the 2 representative images.

### Quantification of soluble cytokines.

Cytokine concentrations in cell-free BAL fluid supernatants were measured via cytokine bead array. All samples were assayed with the LEGENDplex predefined mouse inflammation panel (BioLegend), according to manufacturer directions and analyzed on an LSR II cytofluorometer (Becton, Dickinson, BD) or AURORA spectral cytometer (Cytek). Data were processed with the LEGENDplex online data analysis software suite to determine individual analyte concentrations. Concentrations were multiplied by total BAL fluid volume collected to obtain total picogram (pg) of protein.

### Quantitative real-time PCR.

Quantitative real-time reverse transcriptase PCR was performed to determine the relative expression of select *Achromobacter* genes. Custom PrimeTime qPCR probe assays were designed and validated for genes of interest. DNA-free RNA was isolated and purified using TRIzol reagent according to the manufacturer’s instructions. cDNA libraries were subsequently generated with the High-Capacity RNA-to-cDNA kit (Invitrogen). Quantitative PCR was carried out with PrimeTime gene expression 2× master mix (Integrated DNA Technologies) on a CFX96 real-time thermocycler (Bio-Rad). Relative expression (transcript) was calculated by the delta-Ct method (37). Samples were normalized to *gyrB* and 16s rRNA transcript.

### Statistical analysis.

Statistical analysis and data visualization was performed with GraphPad Prism 9.5.0. Student’s unpaired t test and ordinary one-way ANOVA with Fisher’s least significant difference (LSD) test were used to assess pairwise and multigroup statistics, respectively. A *P* value less than or equal to 0.05 was considered statistically significant.

## References

[1] Yabuuchi E, Oyama A. 1971. Achromobacter xylosoxidans n. sp. from human ear discharge. Jpn J Microbiol 15:477–481. doi:10.1111/j.1348-0421.1971.tb00607.x. 5316576

[2] Neidhöfer C, Berens C, Parčina M. 2022. An 18-year dataset on the clinical incidence and MICs to antibiotics of Achromobacter spp. (labeled biochemically or by MAL-DI-TOF MS as A. xylosoxidans), largely in patient groups other than those with CF. Antibiotics (Basel) 11:311. doi:10.3390/antibiotics11030311. 35326774 PMC8944543

[3] Bonis BM, Hunter RC. 2022. JMM Profile: Achromobacter xylosoxidans: the cloak-and-dagger opportunist. J Med Microbiol 71. doi:10.1099/jmm.0.001505. 35587447

[4] Veschetti L, Boaretti M, Saitta GM, Passarelli Mantovani R, Lleò MM, Sandri A, Malerba G. 2022. Achromobacter spp. prevalence and adaptation in cystic fibrosis lung infection. Microbiol Res 263:127140. doi:10.1016/j.micres.2022.127140. 35931003

[5] Esposito S, Pisi G, Fainardi V, Principi N. 2021. What is the role of Achromobacter species in patients with cystic fibrosis? Front Biosci (Landmark Ed) 26:1613–1620. doi:10.52586/5054. 34994175

[6] Li X, Hu Y, Gong J, Zhang L, Wang G. 2013. Comparative genome characterization of Achromobacter members reveals potential genetic determinants facilitating the adaptation to a pathogenic lifestyle. Appl Microbiol Biotechnol 97:6413–6425. doi:10.1007/s00253-013-5018-3. 23749121

[7] Jakobsen TH, Hansen MA, Jensen P, Hansen L, Riber L, Cockburn A, Kolpen M, Rønne Hansen C, Ridderberg W, Eickhardt S, Hansen M, Kerpedjiev P, Alhede M, Qvortrup K, Burmølle M, Moser C, Kühl M, Ciofu O, Givskov M, Sørensen SJ, Høiby N, Bjarnsholt T. 2013. Complete genome sequence of the cystic fibrosis pathogen Achromobacter xylosoxidans NH44784-1996 complies with important pathogenic phenotypes. PLoS One 8:e68484. doi:10.1371/journal.pone.0068484. 23894309 PMC3718787

[8] Hu Y, Zhu Y, Ma Y, Liu F, Lu N, Yang X, Luan C, Yi Y, Zhu B. 2015. Genomic insights into intrinsic and acquired drug resistance mechanisms in Achromobacter xylosoxidans. Antimicrob Agents Chemother 59:1152–1161. doi:10.1128/AAC.04260-14. 25487802 PMC4335856

[9] Jeukens J, Freschi L, Vincent AT, Emond-Rheault JG, Kukavica-Ibrulj I, Charette SJ, Levesque RC. 2017. A pan-genomic approach to understand the basis of host adaptation in achromobacter. Genome Biol Evol 9:1030–1046. doi:10.1093/gbe/evx061. 28383665 PMC5405338

[10] Filipic B, Malesevic M, Vasiljevic Z, Lukic J, Novovic K, Kojic M, Jovcic B. 2017. Uncovering differences in virulence markers associated with Achromobacter species of CF and non-CF origin. Front Cell Infect Microbiol 7:224. doi:10.3389/fcimb.2017.00224. 28611955 PMC5447083

[11] Veschetti L, Sandri A, Johansen HK, Lleò MM, Malerba G. 2020. Hypermutation as an Evolutionary Mechanism for Achromobacter xylosoxidans in Cystic Fibrosis Lung Infection. Pathogens 9:72. doi:10.3390/pathogens9020072. 31973169 PMC7168687

[12] Veschetti L, Sandri A, Patuzzo C, Melotti P, Malerba G, Lleo MM. 2021. Genomic characterization of Achromobacter species isolates from chronic and occasional lung infection in cystic fibrosis patients. Microb Genom 7. 10.1099/mgen.0.000606 PMC8477391 34292148

[13] Veschetti L, Sandri A, Patuzzo C, Melotti P, Malerba G, Lleò MM. 2021. Mobilome analysis of Achromobacter spp. isolates from chronic and occasional lung infection in cystic fibrosis patients. Microorganisms 9:130. doi:10.3390/microorganisms9010130. 33430044 PMC7826576

[14] Gabrielaite M, Bartell JA, Nørskov-Lauritsen N, Pressler T, Nielsen FC, Johansen HK, Marvig RL. 2021. Transmission and antibiotic resistance of Achromobacter in cystic fibrosis. J Clin Microbiol 59. doi:10.1128/JCM.02911-20. PMC8092725 33472899

[15] Khademi SMH, Gabrielaite M, Paulsson M, Knulst M, Touriki E, Marvig RL, Påhlman LI. 2021. Genomic and phenotypic evolution of Achromobacter xylosoxidans during chronic airway infections of patients with cystic fibrosis. mSystems 6:e0052321. doi:10.1128/mSystems.00523-21. 34184916 PMC8269239

[16] Ormerod KL, George NM, Fraser JA, Wainwright C, Hugenholtz P. 2015. Comparative genomics of non-pseudomonal bacterial species colonising paediatric cystic fibrosis patients. PeerJ 3:e1223. doi:10.7717/peerj.1223. 26401445 PMC4579023

[17] Le Goff M, Vastel M, Lebrun R, Mansuelle P, Diarra A, Grandjean T, Triponney P, Imbert G, Gosset P, Dessein R, Garnier F, Durand E. 2022. Characterization of the Achromobacter xylosoxidans rtype VI secretion system and its implication in cystic fibrosis. Front Cell Infect Microbiol 12:859181. doi:10.3389/fcimb.2022.859181. 35782124 PMC9245596

[18] Pickrum AM, DeLeon O, Dirck A, Tessmer MH, Riegert MO, Biller JA, Ledeboer NA, Kirby JR, Frank DW. 2020. Achromobacter xylosoxidans cellular pathology is correlated with activation of a Type III secretion system. Infect Immun 88. doi:10.1128/IAI.00136-20. PMC7309624 32366575

[19] Pickrum AM, Riegert MO, Wells C, Brockman K, Frank DW. 2022. The in vitro replication cycle of Achromobacter xylosoxidans and identification of virulence genes associated with cytotoxicity in macrophages. Microbiol Spectr 10:e0208322. doi:10.1128/spectrum.02083-22. 35856670 PMC9430717

[20] Hyodo S, Katahira S, Shigeta S. 1982. Experimental infection of immunocompromised mice with Achromobacter xylosoxidans. Tohoku J Exp Med 136:251–261. doi:10.1620/tjem.136.251. 6803396

[21] Prado MKB, Locachevic GA, Zoccal KF, Paula-Silva FWG, Fontanari C, Ferreira JC, Pereira PAT, Gardinassi LG, Ramos SG, Sorgi CA, Darini ALC, Faccioli LH. 2017. Leukotriene B(4) is essential for lung host defence and alpha-defensin-1 production during Achromobacter xylosoxidans infection. Sci Rep 7:17658. doi:10.1038/s41598-017-17993-9. 29247243 PMC5732241

[22] Elias-Oliveira J, Prado MKB, Souza COS, Pastore MR, Ramos SG, Costa Darini AL, Gardinassi LG, Faccioli LH. 2022. CD14 signaling mediates lung immunopathology and mice mortality induced by Achromobacter xylosoxidans. Inflamm Res 71:1535–1546. doi:10.1007/s00011-022-01641-8. 36280620 PMC9592541

[23] Rosen BH, Chanson M, Gawenis LR, Liu J, Sofoluwe A, Zoso A, Engelhardt JF. 2018. Animal and model systems for studying cystic fibrosis. J Cyst Fibros 17:S28–S34. doi:10.1016/j.jcf.2017.09.001. 28939349 PMC5828943

[24] Turton KB, Ingram RJ, Valvano MA. 2021. Macrophage dysfunction in cystic fibrosis: nature or nurture? J Leukoc Biol 109:573–582. doi:10.1002/JLB.4RU0620-245R. 32678926

[25] Bonfield TL, Hodges CA, Cotton CU, Drumm ML. 2012. Absence of the cystic fibrosis transmembrane regulator (Cftr) from myeloid-derived cells slows resolution of inflammation and infection. J Leukoc Biol 92:1111–1122. doi:10.1189/jlb.0412188. 22859830 PMC3476241

[26] Bragonzi A. 2010. Murine models of acute and chronic lung infection with cystic fibrosis pathogens. Int J Med Microbiol 300:584–593. doi:10.1016/j.ijmm.2010.08.012. 20951086

[27] Gosselin D, Stevenson MM, Cowley EA, Griesenbach U, Eidelman DH, Boule M, Tam MF, Kent G, Skamene E, Tsui LC, Radzioch D. 1998. Impaired ability of Cftr knockout mice to control lung infection with Pseudomonas aeruginosa. Am J Respir Crit Care Med 157:1253–1262. doi:10.1164/ajrccm.157.4.9702081. 9563748

[28] Heeckeren A, Walenga R, Konstan MW, Bonfield T, Davis PB, Ferkol T. 1997. Excessive inflammatory response of cystic fibrosis mice to bronchopulmonary infection with Pseudomonas aeruginosa. J Clin Invest 100:2810–2815. doi:10.1172/JCI119828. 9389746 PMC508486

[29] Yadav A, Saini V, Arora S. 2010. MCP-1: chemoattractant with a role beyond immunity: a review. Clin Chim Acta 411:1570–1579. doi:10.1016/j.cca.2010.07.006. 20633546

[30] Shi Y, Liu CH, Roberts AI, Das J, Xu G, Ren G, Zhang Y, Zhang L, Yuan ZR, Tan HS, Das G, Devadas S. 2006. Granulocyte-macrophage colony-stimulating factor (GM-CSF) and T-cell responses: what we do and don't know. Cell Res 16:126–133. doi:10.1038/sj.cr.7310017. 16474424

[31] Deshmane SL, Kremlev S, Amini S, Sawaya BE. 2009. Monocyte chemoattractant protein-1 (MCP-1): an overview. J Interferon Cytokine Res 29:313–326. doi:10.1089/jir.2008.0027. 19441883 PMC2755091

[32] Serbina NV, Kuziel W, Flavell R, Akira S, Rollins B, Pamer EG. 2003. Sequential MyD88-independent and -dependent activation of innate immune responses to intracellular bacterial infection. Immunity 19:891–901. doi:10.1016/s1074-7613(03)00330-3. 14670305

[33] Ramirez-Alejo N, Santos-Argumedo L. 2014. Innate defects of the IL-12/IFN-gamma axis in susceptibility to infections by mycobacteria and salmonella. J Interferon Cytokine Res 34:307–317. doi:10.1089/jir.2013.0050. 24359575 PMC4015507

[34] Car BD, Eng VM, Schnyder B, LeHir M, Shakhov AN, Woerly G, Huang S, Aguet M, Anderson TD, Ryffel B. 1995. Role of interferon-gamma in interleukin 12-induced pathology in mice. Am J Pathol 147:1693–1707. 7495294 PMC1869961

[35] Van Hoecke L, Job ER, Saelens X, Roose K. 2017. Bronchoalveolar lavage of murine lungs to analyze inflammatory cell infiltration. JoVE. doi:10.3791/55398. PMC5607888 28518083

[36] Davenport ML, Sherrill TP, Blackwell TS, Edmonds MD. 2020. Perfusion and inflation of the mouse lung for tumor histology. JoVE. doi:10.3791/60605. PMC7877253 32831298

[37] Livak KJ, Schmittgen TD. 2001. Analysis of relative gene expression data using real-time quantitative PCR and the 2(-Delta Delta C(T)) Method. Methods 25:402–408. doi:10.1006/meth.2001.1262. 11846609

