# Supplementary material for: Identification of Virulence Factors Involved in a Murine Model of Severe Achromobacter xylosoxidans Infection
Source: Infect Immun. 2023 May 31;91(7):e00037-23. doi: 10.1128/iai.00037-23 (PMC10353400; doi:10.1128/iai.00037-23)
Supplement: Supplemental file 1 — Fig. S1 and S2. Download iai.00037-23-s0001.pdf, PDF file, 0.3 MB [file iai.00037-23-s0001.pdf]

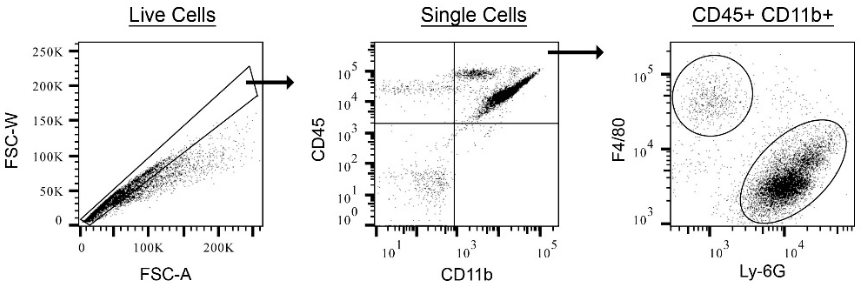

Figure S1. BAL cell gating strategy

Gating strategy used to determine macrophages (CD45+ CD11b+ F4/80+) and neutrophils (CD45+ CD11b+ Ly-6G+).

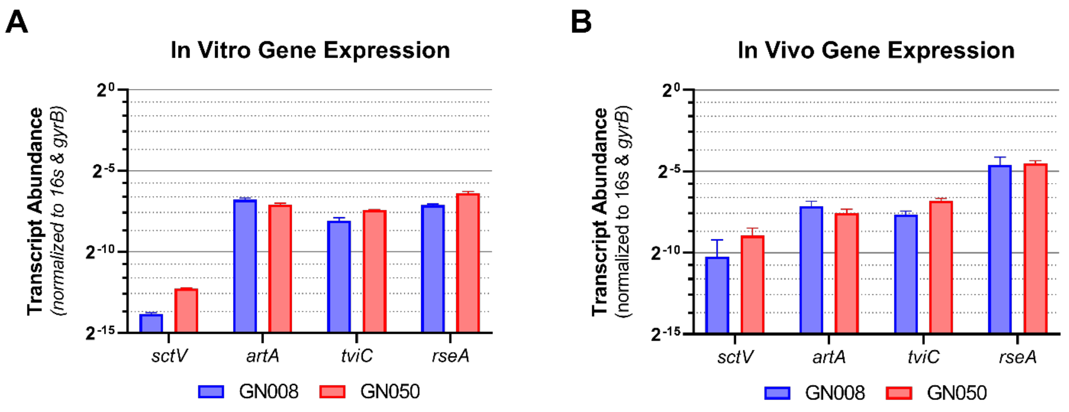

Figure S2. Expression of AX genes in vitro and in vivo

RT-qPCR was performed on AX cultured in LB broth (A) or collected from the lungs of C57Bl/6 24 h after infection (B). Transcripts of *sctV*, *artA*, *tviC*, and *rseA* were detected in both GN008 and GN050 under *in vitro* and *in vivo* conditions. Transcript abundance was normalized to *gyrB* and 16s rRNA. n = 3 for *in vitro*, n = 4 for *in vivo* samples.
